# Central precocious puberty as the initial manifestation of multisystem involvement caused by *de novo* heterozygous *KMT2B* mutation and *STS* hemizygous deletion: a case report

**DOI:** 10.3389/fendo.2026.1869758

**Published:** 2026-06-17

**Authors:** Yaqin Feng, Li Yang, Qing-Bo Xu, Lan-Fang Cao

**Affiliations:** Jiangxi Provincial Children's Hospital; JXHC Key Laboratory of Precise Prevention and Treatment for Childhood Diabetes, Nanchang, Jiangxi, China

**Keywords:** *KMT2B*, *STS*, central precocious puberty, X-linked ichthyosis, pediatric endocrinology

## Abstract

**Background:**

Pathogenic loss-of-function variants in the *KMT2B* gene cause a rare autosomal dominant disorder with two major phenotypes: *KMT2B*-related dystonia (DYT-*KMT2B*) and *KMT2B*-related neurodevelopmental disorder (*KMT2B*-NDD). Central precocious puberty (CPP) has been documented as a comorbidity in patients with DYT-*KMT2B*, but has not been reported as the primary clinical manifestation of the disorder in patients without dystonia. Deletions or pathogenic variants in the *STS* gene cause X-linked ichthyosis (XLI). Concurrent *KMT2B* and *STS* alterations have not been well characterised, and GnRH analogue therapy for CPP in *KMT2B*-related disorders has not been previously described.

**Case presentation:**

An 8-year-10-month-old male presented with a 1-2−year history of progressive increase in penile length and girth. Physical examination revealed distinctive craniofacial dysmorphism, classic ichthyotic scaling, pubertal changes consistent with precocious puberty, and normal muscle tone without dystonic features. Biochemical testing confirmed markedly elevated serum total testosterone, a positive gonadotropin-releasing hormone (GnRH) stimulation test consistent with CPP, and bone age advanced by 1.2 years relative to chronological age. Neurocognitive assessment identified mild intellectual disability. Family-based whole-exome sequencing and copy number variation (CNV) analysis identified a *de novo* heterozygous pathogenic nonsense variant in *KMT2B* (NM_014727.3: c.4213del, p.Leu1405Ter) and a hemizygous pathogenic full-coding deletion of *STS*. The patient was treated with leuprorelin acetate for CPP and topical emollients for XLI. At 3−month follow−up, physical signs of puberty remained stable. GnRHa stimulation test showed indicating but incomplete suppression of the hypothalamic-pituitary-gonadal axis. The ichthyotic skin lesions improved markedly with topical therapy. The leuprorelin acetate dose was subsequently increased. The patient is continuing on the current regimen with close monitoring.

**Conclusion:**

Precocious puberty has been reported in eleven patients with *KMT2B*−related dystonia. This case documents the co−occurrence of a *KMT2B*−related disorder and XLI in a patient, and provides documentation of GnRH analogue therapy and structured endocrine follow−up for CPP in this population. *KMT2B* haploinsufficiency may contribute to premature activation of the hypothalamic−pituitary−gonadal axis through disruption of ERα−mediated transcriptional regulation and altered epigenetic programming at key hypothalamic developmental genes. Clinically, children with *KMT2B*−related disorders should undergo comprehensive endocrine assessment to facilitate early diagnosis and timely intervention.

## Introduction

The lysine-specific methyltransferase 2B gene (*KMT2B*, OMIM: 606834), located on chromosome 19q13.12, encodes a histone lysine methyltransferase that catalyzes H3K4 methylation, a key epigenetic modification regulating gene expression during embryonic development, cellular differentiation, and central nervous system homeostasis (1) Loss-of-function variants in *KMT2B* cause two overlapping autosomal dominant conditions: *KMT2B*-related dystonia(DYT-*KMT2B*,OMIM 617284) and *KMT2B*-related neurodevelopmental disorder (*KMT2B*-related NDD,OMIM 619934) (2). The classic clinical phenotype is defined by progressive generalized dystonia with onset in childhood, often accompanied by non-motor features including intellectual disability, microcephaly, distinctive craniofacial dysmorphism, and occasional endocrine abnormalities (2–5). Atypical presentations, particularly those dominated by endocrine manifestations such as central precocious puberty without overt dystonia, remain poorly described in the existing literature.

The steroid sulfatase gene (*STS*, OMIM: 300747), mapped to chromosome Xp22.31, encodes steroid sulfatase, a rate-limiting enzyme in cholesterol sulfate metabolism that is critical for epidermal barrier function and steroid hormone precursor processing (6). Hemizygous deletions or loss-of-function variants in *STS* cause X-linked ichthyosis (XLI, OMIM 308100), an X-linked recessive genodermatosis with a male prevalence of 1 in 6000 to 1 in 2000 (7). Affected males typically present with generalized dry, scaly skin in infancy, and may also develop extra-cutaneous manifestations including corneal opacities, cryptorchidism, and neurodevelopmental deficits (7–9).

Digenic pathogenic variants can lead to complex, overlapping clinical phenotypes that pose substantial diagnostic challenges for clinicians. To date, *KMT2B*-related disorders and XLI have been characterized as distinct monogenic conditions, with limited data on their co-occurrence in a single patient. Approximately 298 patients with *KMT2B*-related disorders have been documented worldwide, including 271with DYT-*KMT2B* and 27 with the *KMT2B*-related NDD (2, 3, 10–13). Most research focusing on the classic motor phenotype of progressive dystonia. While precocious puberty has been reported as a systemic feature in several patients with *KMT2B*−related dystonia, it has not been previously described in a patient with the *KMT2B*-NDD phenotype. This report describes a pediatric patient with a *de novo* heterozygous pathogenic *KMT2B* variant and a hemizygous *STS* deletion, presented with central precocious puberty, low birth weight, feeding difficulties, developmental delay, and mild intellectual disability, and XLI. This case expands the genotypic of KMT2B−related disorders, and provides documentation of GnRH analogue therapy and structured endocrine follow−up for CPP in this population.

## Case report

An 8-year-10-month-old Chinese boy was referred to the Department of Endocrinology and Genetic Metabolism, Jiangxi Children’s Hospital in November 2025 after his parents had noted a progressive increase in his penile length and girth over the preceding 1–2 years. He was the second child of non-consanguineous parents, delivered via cesarean section at 39 weeks of gestation with a birth weight of 2 kg. No perinatal asphyxia was reported, but feeding difficulties were present in the infancy, and global developmental delay was noted throughout early childhood, with persistent growth parameters below the mean for age-matched peers. He had a history of recurrent upper respiratory tract infections in early childhood, but no history of febrile seizures, epilepsy, or other significant medical events. At presentation, he could recognise only simple characters; his speech was fluent but poorly articulated, and occasional oropharyngeal choking was noted. No tiptoe walking, headache, vomiting, or café-au-lait spots were reported. Generalized skin dryness and scaling had been present since infancy, predominantly affecting the neck and extensor surfaces of the lower limbs, with no pruritus or clear exacerbating or relieving factors. Family history was notable for ichthyosis in the maternal grandfather, with no available clinical details; the patient’s father (163 cm) and mother (153 cm) were healthy, with no history of developmental delay, skin disease, or endocrine disorders, and his older brother had normal growth and development with no skin abnormalities.

**Figure 1:**
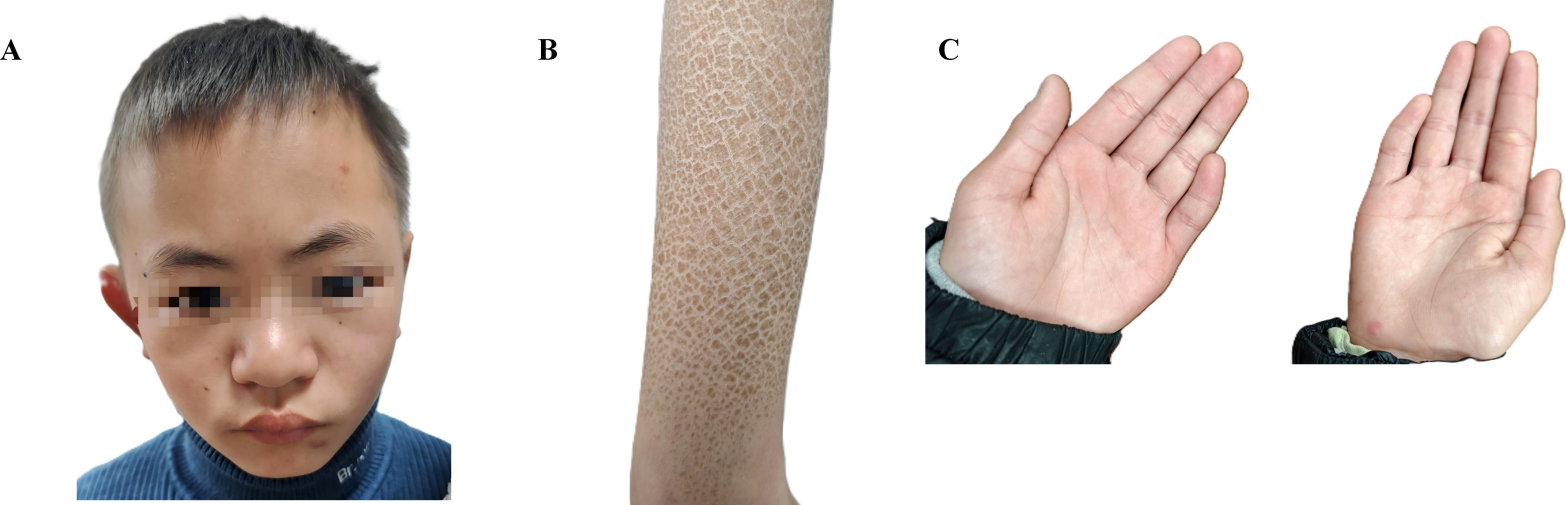
Clinical phenotypic manifestations of the proband with concurrent *KMT2B*-related disorder and X-linked ichthyosis (XLI). **(A)** Facial photograph of the patient, demonstrating the distinctive craniofacial dysmorphic features associated with *KMT2B*-related disorder, including a broad forehead, bulbous nose, large auricles, micrognathia, wide mouth, and thick upper lip. The periorbital region has been pixelated to protect the patient’s privacy, in full compliance with medical publication ethics requirements. **(B)** Cutaneous lesions on the extensor surface of the patient’s upper limb, showing the characteristic reticular, polygonal, adherent scaling pathognomonic of XLI caused by *STS* gene deletion. **(C)** Bilateral palms of the patient, showing inward curving of both fifth fingers (clinodactyly), with no significant scaling, hyperkeratosis, or abnormal texture, consistent with the typical sparing of palmoplantar surfaces in XLI.

Physical examination at presentation revealed a head circumference of 48 cm, height of 127.5 cm (–1.23 standard deviation for age-and sex-matched peers), and weight of 23 kg, with a normal body habitus. He had distinctive craniofacial dysmorphism, including a broad forehead, bulbous nose, large low-set ears, micrognathia, wide mouth, thick upper lip, and widely spaced teeth (**Figure 1A**). Ophthalmic inspection showed no corneal opacities. Cutaneous examination revealed generalized xerosis with adherent ichthyotic scaling, most severe on the neck and upper limb extensor surfaces (**Figure 1B**). He had minimal laryngeal prominence, no facial hair, and bilateral breasts at Tanner stage 1 with no nipple inversion. Cardiopulmonary and abdominal examinations were unremarkable, with no hepatosplenomegaly. Musculoskeletal examination showed bilateral clinodactyly of the fifth fingers (**Figure 1C**), with normal muscle strength and tone in all four limbs, no dystonic posturing, and a normal gait. Genitourinary examination showed Tanner stage 1 pubic hair, stretched penile length (SPL) of 7.0 cm, and penile circumference of 6.5 cm, and bilateral testes palpable in the scrotum with a volume of approximately 5 ml each.

A gonadotropin−releasing hormone (GnRH) stimulation test was performed using intravenous gonadorelin at a dose of 2.5 μg/kg.Blood samples for LH and FSH were drawn at baseline and at 30, 60, 90 and 120 minutes after injection.The test showed a basal luteinizing hormone (LH) level of 1.95mIU/mL (reference range:0.05-1.44mIU/mL), basal follicle-stimulating hormone (FSH) level of 2.82mIU/mL (reference range:0.43-7.14mIU/mL), a peak LH level of 47.37 mIU/mL, a peak FSH level of 12.63mIU/mL, and a peak LH/FSH ratio of 6.75 (diagnostic threshold for central precocious puberty:peak LH ≥5 mIU/mL and LH/FSH>0.6), consistent with a central etiology of precocious puberty. The total testosterone level was 319.86 ng/dL (reference for prepubertal males:0-25.07ng/dL), which was markedly elevated. Androstenedione was 0.33 ng/mL (reference range:0.7-3.6ng/mL), which was decreased, and dehydroepiandrosterone sulfate was 34.60 μg/dL (reference range:80-560μg/dL), which was also decreased. Estradiol, progesterone, and prolactin were all within their respective normal limits. Anti−Müllerian hormone was 20.52 ng/mL (reference range: 38.79-294.19ng/mL), which was decreased; inhibin B was 217.58 pg/mL (reference range: 27.06-48.96 pg/mL), which was elevated; and inhibin A was 4.14 pg/mL (reference range: 1.0-2.2pg/mL), which was also elevated. Thyroid function testing showed a free thyroxine level of 0.82 ng/mL (reference range:0.828-1.494ng/mL), with thyroid−stimulating hormone (reference range: 0.58-5.39 mIU/L) and free triiodothyronine (reference range: 2.585-5.098 pg/mL) both within the normal range. Serum alkaline phosphatase was 801 U/L (reference range: 143–406 U/L), which was elevated, whereas serum calcium, phosphorus, 25−hydroxyvitamin D, insulin−like growth factor 1, and hepatic and renal function were all within their respective normal limits. Chromosomal karyotype analysis confirmed a normal 46, XY male karyotype.

Imaging studies were performed to rule out secondary causes of precocious puberty and characterize systemic involvement. Bone age was assessed independently by two endocrinologists using the Greulich−Pyle method and was read as 10.0 years, 1.2 years advanced relative to chronological age. Electrocardiogram showed sinus arrhythmia and left ventricular high voltage. Testicular ultrasound revealed normal parenchymal structure, with testicular volumes of 5.79 ml (left) and 5.24 ml (right). Three-hour video electroencephalogram, cranial and pituitary magnetic resonance imaging, echocardiography, and abdominal and urinary tract ultrasound showed no structural or functional abnormalities. Neurodevelopmental assessment using the Chinese Wechsler Intelligence Scale for Children showed a verbal IQ of 75, performance IQ of 68, and full-scale IQ of 69, consistent with mild intellectual disability.

**Figure 2:**
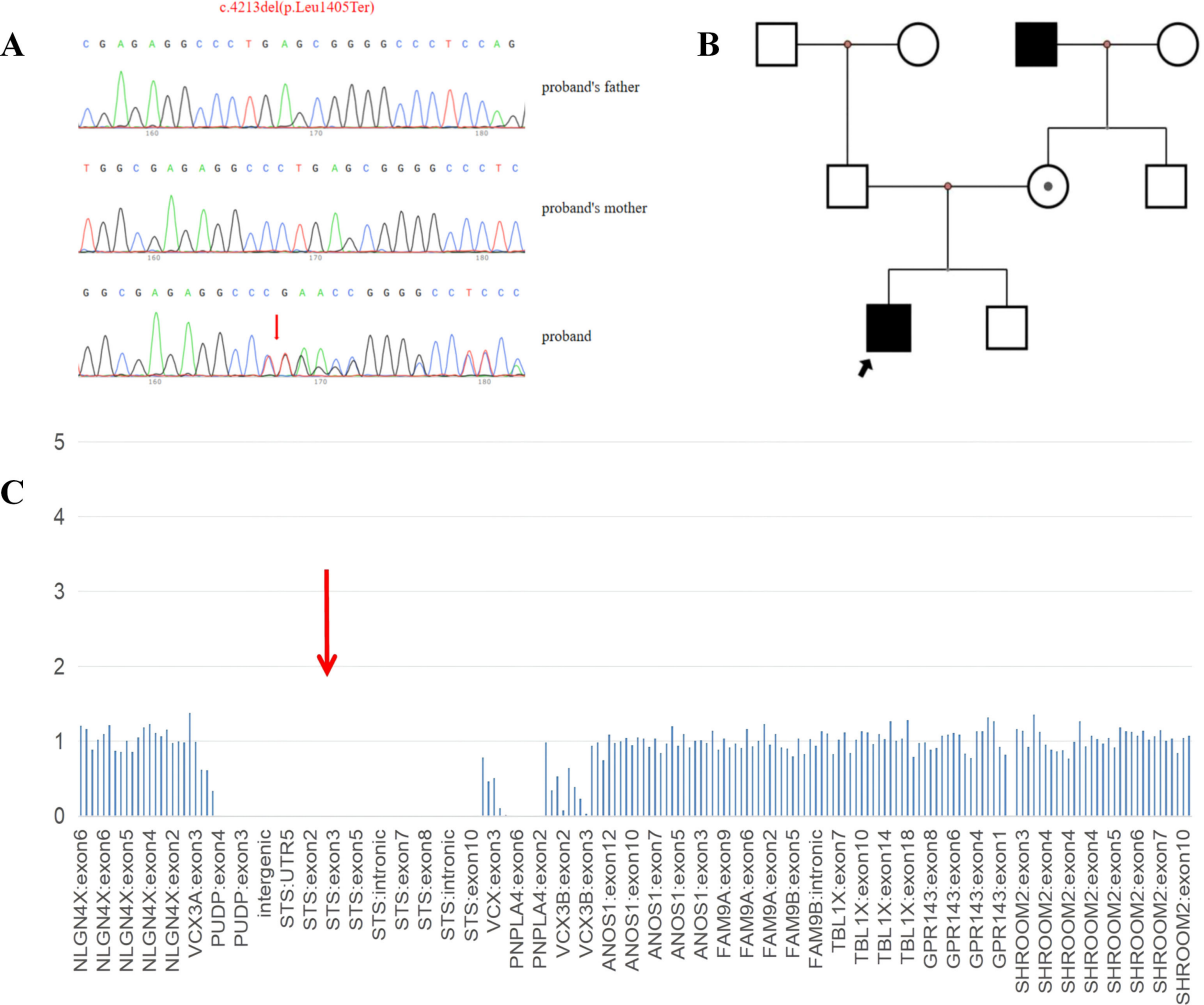
Molecular genetic findings of the proband. **(A)** Sanger sequencing validation of the *KMT2B* variant. Sequencing chromatograms show the *de novo* heterozygous loss-of-function variant NM_014727.3:c.4213del (p.Leu1405Ter) in the proband, with wild-type sequences confirmed in both unaffected parents. The red vertical line marks the position of the single nucleotide deletion. **(B)** Family pedigree for the X-linked inheritance of the *STS* gene deletion. Squares represent male individuals, circles represent female individuals; filled black symbols indicate male individuals affected with X-linked ichthyosis caused by hemizygous *STS* deletion; symbols with a central dot indicate heterozygous female carriers of the *STS* deletion. The black arrow indicates the male proband, who inherited the hemizygous full-coding *STS* deletion from his heterozygous carrier mother. **(C)** Copy number variation (CNV) analysis from whole-exome sequencing data. The y-axis represents normalized genomic copy number, and the x-axis represents genes and corresponding exons in the Xp22.31 region. The red arrow indicates the complete deletion of all coding exons of the *STS* gene in the proband (copy number = 0, consistent with a hemizygous deletion in a male individual), with normal copy number of all flanking genes.

Given the patient’s complex multisystem presentation, genetic testing was deemed clinically indicated. After obtaining written informed consent from the patient’s legal guardians, peripheral blood samples were collected from the patient and both parents for family-based whole-exome sequencing (WES) and copy number variation (CNV) analysis. A *de novo* heterozygous nonsense variant c.4213del (p.Leu1405Ter) was identified in the *KMT2B* gene (NM_014727.3) (**Figure 2A**). The variant is absent from public population databases including ClinVar, ChinaMap, and gnomAD, and introduces a premature termination codon in exon 16 of the 37-exon *KMT2B* transcript. Given its position well upstream of the final exon-exon junction, the mutant transcript is predicted to undergo nonsense-mediated mRNA decay (NMD). Even if a fraction of the transcript were to escape NMD and be translated into a C-terminally truncated protein lacking the SET domain, the consequence would remain loss of *KMT2B* methyltransferase function.This variant was classified as pathogenic per American College of Medical Genetics and Genomics (ACMG) guidelines (PVS1+PS2_Supporting+PM2_Supporting); the PVS1 criterion was applied because the nonsense variant in exon 16 is predicted to trigger NMD, leading to a loss-of-function effect. CNV analysis identified a hemizygous deletion spanning exons 1–11 of the *STS* gene (**Figure 2C**); family pedigree analysis confirmed this deletion was maternally inherited, with the patient’s mother being a heterozygous carrier and the maternal grandfather presenting with ichthyosis, consistent with the X-linked recessive inheritance pattern of XLI (**Figure 2B**). This *STS* deletion was classified as pathogenic per ACMG guidelines (PVS1+PS4_VeryStrong+PM2_Supporting+PP4). The *KMT2B* c.4213del variant was confirmed by Sanger sequencing. For the STS deletion, multiplex ligation-dependent probe amplification(MLPA) was performed using a probe mix targeting all exons of the STS gene, which confirmed the hemizygous deletion in the proband.

Based on the patient’s clinical manifestations, laboratory and imaging findings, and genetic testing results, the final diagnoses were (1): *KMT2B*-related disorder (2); X-linked ichthyosis (3); central precocious puberty (4); mild intellectual disability. From January 25, 2026, the patient received subcutaneous injections of leuprorelin acetate microspheres 3 mg every 28 days for the treatment of central precocious puberty. He was referred to the dermatology department for ongoing management of XLI, with a regimen of daily topical emollients to improve skin hydration and reduce scaling. Long-term neurological follow-up was arranged to monitor for the emergence or progression of dystonia, a core feature of *KMT2B*-related disease. Comprehensive genetic counseling was provided to the family, including information on the inheritance patterns of both variants and recurrence risk for future pregnancies. The patient returned for his follow-up evaluation on April 21, 2026, approximately three months after the initiation of gonadotropin-releasing hormone analog therapy. On physical examination at the 3-month visit, height was 132.4 cm (height standard deviation score [HtSDS] −0.73 for age-and sex-matched peers), with a growth velocity of 11.8 cm/year over the preceding 5 months. The HtSDS improved from −1.23 at baseline to −0.73 at the most recent assessment. Stretched penile length(SPL) was 7 cm, penile circumference was 6.5 cm, and testicular volume was approximately 5 ml bilaterally, all unchanged from the initial measurements obtained at diagnosis. Pubic hair remained Tanner stage 1 and axillary hair remained A1, indicating that clinical progression of puberty had been halted. A GnRH stimulation test performed on the same day yielded the following results: basal luteinizing hormone was 0.67 mIU/ml (reference range: 0.02-0.3mIU/mL), basal follicle-stimulating hormone was 1.83 mIU/mL (reference range: 0.26-3.0 mIU/mL), peak LH was 3.99 mIU/mL, and peak FSH was 3.31 mIU/mL. Total testosterone had declined from 319.86 ng/dl at baseline to 81.83 ng/dL (reference range: 7-29.44ng/dL), and estradiol was 13.35 pg/mL (reference range: 4.9-10.9pg/mL). Hepatic and renal function, cardiac enzymes, serum lipids, and fasting blood glucose were all within their respective normal limits. Given the incomplete biochemical suppression, the leuprorelin acetate microspheres dose was increased from 3 mg to 3.75 mg every 28 days. At the most recent visit on May 19, 2026, the patient’s height was 133.0 cm and weight was 24.5 kg.The patient is continuing on the current regimen with close monitoring, and a further GnRH stimulation test will be performed at the next follow−up visit to reassess biochemical suppression. A timeline of the patient’s key clinical milestones, diagnoses, and treatments is provided in **Table 1**.

**Table 1:** Timeline of key clinical events for the patient.

Time point (age)	Key clinical events
0-1years	Feeding difficulties noted; onset of persistent skin dryness and scaling
1-5years	Global developmental delay identified; recurrent upper respiratory tract infections; progressive ichthyotic skin changes
6–7 years	Parents first noted a progressive increase in the patient’s penile length and girth
8 years 10 months	Initial clinical presentation for precocious puberty; comprehensive laboratory, imaging, and neurodevelopmental assessment; genetic testing initiated
9 years 0 months	Molecular diagnosis confirmed; initiation of leuprorelin acetate therapy for central precocious puberty; dermatology referral and topical emollient therapy for X-linked ichthyosis
9 years 3 months	Follow-up at 3 months of GnRHa therapy: height 132.4 cm (−0.73HtSDS), growth velocity 11.8cm/year; physical signs of puberty remained stable; GnRH stimulation test showed markedly attenuated but incomplete biochemical suppression; leuprorelin acetate dose increased to 3.75 mg every 28 days;skin lesions improved with topical therapy.
9 years 3 months–present	Continued multidisciplinary surveillance of growth, bone maturation, skin condition, and neurological status; long-term monitoring for dystonia

## Patient perspective: guardian’s narrative

As the parents of this boy, we have faced many challenges in seeking care for him over the years. For a long time, we took him to local hospitals for his skin changes and developmental delay, but we never received a clear explanation for his symptoms, and genetic testing was not recommended until we brought him to the pediatric endocrinology department because of a noticeable increase in his penile length and girth. The definitive genetic diagnosis was a pivotal moment for our family, as it finally helped us understand the root cause of our son’s multiple health issues.

The treatment for his precocious puberty has been highly effective, with no obvious side effects, and his skin condition has improved significantly with regular use of moisturizers. We are reassured by the clear follow-up plan to monitor his growth, development, and neurological status long-term. We are deeply grateful for the exceptional care our son has received, and we hope that sharing our experience can help other children with similar rare symptoms receive an earlier diagnosis and appropriate treatment.

## Discussion

The clinical presentation of this patient reflects the combined effects of two distinct monogenic disorders, resulting in a complex multisystem phenotype involving the endocrine, neurological, craniofacial, and cutaneous systems. The overlapping features of *KMT2B*-related disorders and XLI create a diagnostic challenge, as no single gene variant can fully explain all of the patient’s clinical manifestations. This case highlights the critical importance of comprehensive genomic testing, including both sequencing and CNV analysis, in the evaluation of children with unexplained multisystem disease, particularly when clinical features span multiple traditionally distinct disease categories.

*KMT2B*-related disorders are defined by marked phenotypic heterogeneity, with a wide spectrum of clinical presentations ranging from isolated progressive dystonia to global neurodevelopmental impairment without motor manifestations (2, 10–12). DYT-*KMT2B*, the classic phenotype is dominated by childhood-onset progressive generalized dystonia, typically beginning with lower limb involvement and tiptoe walking before progressing to involve the trunk, upper limbs, and cranial musculature (2). The clinical manifestations of *KMT2B*-NDD are primarily characterized by neurodevelopmental defects, without significant dystonia.Characteristic features include pre- and postnatal growth restriction, feeding difficulties, microcephaly, developmental delay and intellectual disability (particularly speech delay), ectodermal dysplasia, and genital malformations in males (2). The pathogenic *KMT2B* variant identified in this patient, c.4213del (p.Leu1405Ter), introduces a premature termination codon in exon 16 of the 37-exon transcript, which is predicted to trigger nonsense-mediated mRNA decay and result in loss of *KMT2B* function through haploinsufficiency (14). Even if a truncated protein lacking the SET domain were produced, it would be catalytically inactive. This loss of *KMT2B* methyltransferase activity is expected to dysregulate H3K4 methylation and alter the expression of downstream target genes involved in neurodevelopment, craniofacial morphogenesis, and endocrine regulation (15). The patient presented with central precocious puberty, low birth weight, feeding difficulties, developmental delay, and mild intellectual disability. Although his speech was fluent but poorly articulated and occasional oropharyngeal choking was noted, he did not exhibit dysarthria, nasal speech, or other signs of bulbar dysfunction characteristic of DYT-*KMT2B*. His speech difficulties were linguistic in nature and consistent with mild intellectual disability, rather than reflecting motor dysfunction of the articulatory muscles. While the patient has not developed dystonia to date, the variable age of onset of motor manifestations in *KMT2B*-related disease means that delayed onset cannot be excluded. Previous studies have reported that dystonia may emerge as late as adulthood in some patients (16), and that approximately 17% of patients with pathogenic *KMT2B* variants present with neurodevelopmental deficits without dystonia (17). Long-term neurological surveillance is therefore essential for this patient, to enable early detection and intervention for motor manifestations should they emerge.

The patient was diagnosed with CPP because of a progressive increase in penile length and girth over the preceding 1–2 years. To contextualise our case within the existing literature, we reviewed all previously reported *KMT2B* cases with documented endocrine manifestations (**Table 2**). Among these, precocious puberty has been documented in eight patients from the DYT-*KMT2B* subgroup in the largest published cohort, five of whom were male; no cases have been reported in the *KMT2B*-NDD subgroup (5). Among these, precocious puberty was documented in eight patients, all of whom belonged to the DYT−*KMT2B* subgroup; no cases were identified among the nine patients with the *KMT2B*−NDD phenotype, and none of the reported patients presented with CPP as the sentinel manifestation in the absence of dystonia.To our knowledge, CPP has therefore not been previously described as the initial presentation of a *KMT2B*-related disorder in a patient without dystonia. Furthermore, across all reported *KMT2B* cases, we identified no descriptions of GnRHa treatment for precocious puberty or structured endocrine follow-up data in this patient population. We acknowledge that endocrine assessment is not systematically performed in most published *KMT2B* cohorts, and it is possible that mild precocious puberty has been under-recognised.

**Table 2:** KMT2B-related cases with endocrine dysfunction (excluding isolated short stature).

Reference	Year	Phenotypic subgroup	Sex	Age at report	Mutation/Deletion	Endocrine manifestations	Precocious puberty (Y/N)	GnRHa treatment (Y/N)
Faundes V et al. (30)	2017	KMT2B-Related NDD	F	11y	c.1808dupC p.(Leu604Profs*72)	growth hormone deficiency	N	N
Ding S et al. (4)	2024	DYT-KMT2B	M	4y6m	c.8_9insGGCGGCG (p.Ala3Alafs*115)	thyroid dysfunction	N	N
Ding S et al. (4)	2024	DYT-KMT2B	F	15y4m	c.3605dupT (p.Met1202Ilefs*22)	earlier breast development and menstruation, advanced bone age	Y	N
Ding S et al. (4)	2024	DYT-KMT2B	F	20y9m	c.4214dupT (p.Ser1406Glufs*272)	Short stature, advanced bone age,earlier menstruation	Y	N
Cif et al. (5)	2020	DYT-KMT2B	F	22y	c.12_24dup13(p.Ser9Glyfs*111)	Precocious puberty (8 y)	Y	N
Cif et al. (5)	2020	DYT-KMT2B	M	19y	c.188delG(p.Ala40Profs*6)	Precocious puberty (9y)	Y	N
Cif et al. (5)	2022	DYT-KMT2B	M	19y	c.188delG(p.Ala40Profs*6)	Precocious puberty (9y)	Y	N
Cif et al. (5)	2020	DYT-KMT2B	M	26y	c.1107dupC(p.Glu370Argfs*19)	insulin dependent diabetes mellitus,Hypothyroidism	N	N
Cif et al. (5)	2020	DYT-KMT2B	F	29y	c.2137dupA(p.Thr713Asnfs*4)	Precocious puberty	Y	N
Cif et al. (5)	2020	DYT-KMT2B	F	9y	c.3602delC(p.Pro1201Argfs*154)	Precocious puberty	Y	N
Cif et al. (5)	2020	DYT-KMT2B	M	17y	c.3997delG(p.Glu1333Argfs*22)	Precocious puberty,Hypothyroidism	Y	N
Cif et al. (5)	2020	DYT-KMT2B	M	11y	c.4931G>T(p.Cys1644Phe)	Precocious Puberty	Y	N
Cif et al. (5)	2020	DYT-KMT2B	F	44y	c.6439C>T(p.Gln2147)	Hypothyroidism	N	N
Cif et al. (5)	2020	DYT-KMT2B	M	11y6m	c.7348 C>T(p.Arg2450)	Precocious puberty	Y	N
Forzano F et al. (31)	2012	19q13 microdeletion(KMT2B-Related NDD)	F	6y6m	a maternally inherited 3.32 Mbdeletion of chromosome 8q24.21e24.22, a de novo 1.70 Mb deletion of chromo-some 18q22 anda de novo 1.37 Mb deletion of the chromosome 19q13.11e13.12.	Primary hypothyroidism, GH deficiency, partial ACTH deficiency	N	N
Li XY et al. (32)	2020	DYT-KMT2B	F	11y	c.3605dup (p.Met1202Ilefs*22)	earlier breast development and menstruation, advanced bone age	Y	N
Indelicato E et al. (10)	2026	DYT-KMT2B	M	26y	c.2941T>C (p.Cys981Arg)	History of growth hormone therapy	N	N
Present case	2026	Non−dystonic KMT2B−related disorder	M	8y10m	c.4213del (p.Leu1405Ter)	CPP, marginally low FT4	Y	Y

DYT−*KMT2B*, *KMT2B*−related dystonia; *KMT2B*−NDD, *KMT2B*−related neurodevelopmental disorder; CPP, central precocious puberty; GnRHa, gonadotropin−releasing hormone analog; GH, growth hormone; ACTH, adrenocorticotropic hormone; FT4, free thyroxine; M, male; F, female; y, years; m, months.

Isolated short stature, which is a common but nonspecific finding in *KMT2B*−related disorders, was not included in this comparison unless accompanied by other endocrine abnormalities. The present case appears in bold.Non−dystonic *KMT2B*−related disorder

The patient’s central precocious puberty can be understood in the context of the known molecular functions of *KMT2B*. *KMT2B* encodes a histone H3K4 methyltransferase that serves as the catalytic subunit of the *MLL2* complex, responsible for H3K4 trimethylation on specific gene promoters and nearby cis-regulatory sites, where it regulates bivalent developmental genes as well as stem cell and germinal cell differentiation gene sets (14). In addition, *KMT2B* functions as a selective transcriptional coactivator for estrogen receptor alpha: the *MLL2* complex was identified as an ERα coactivator through direct ligand-dependent binding, and *KMT2B* is recruited by ERα to target gene promoters where it catalyses H3K4 methylation to activate transcription; its depletion disrupts oestrogen signalling and alters the expression of multiple ERα-responsive genes (18). Consistent with a role in reproductive axis function, *KMT2B* is highly expressed in the brain, testis, and ovary (14). In the male germline, conditional deletion of *Mll2* leads to a complete block of spermatogenesis and progressive loss of spermatogonia (19). In the female germline, *MLL2* is required in oocytes for bulk H3K4 trimethylation and transcriptional silencing, with *Mll2* deficiency resulting in anovulation and oocyte death (20); conditional deletion of Kmt2b in granulosa cells similarly leads to decreased ovarian follicles, elevated serum FSH levels, and reduced fertility (21). Importantly, loss-of-function *KMT2B* variants cause a distinctive genome-wide DNA hypermethylation profile, with non-random hypermethylation selectively involving promoters and regulatory regions that positively control gene expression (15), which may preferentially silence genes that maintain the prepubertal brake on the HPG axis. Approximately 38% of individuals with *KMT2B*-related disorders have systemic features possibly related to the endocrine system, including precocious puberty (2). We therefore propose that *KMT2B* haploinsufficiency leads to premature activation of the HPG axis through combined disruption of ERα-mediated transcriptional regulation and altered epigenetic programming at key hypothalamic developmental genes. The precise identity of the *KMT2B* target genes in hypothalamic GnRH neurons that mediate this effect remains to be experimentally defined.

The marginally low free thyroxine (0.82 ng/ml) with normal TSH observed in this patient is consistent with the mild endocrine dysfunction recognised in *KMT2B*-related disorders discussed above. Given that the TSH remained normal and the deviation was minimal, no specific intervention was required, and thyroid function will be monitored during follow-up.

In contrast to the epigenetic regulatory dysfunction caused by the *KMT2B* variant, the patient’s cutaneous manifestations are directly attributable to the hemizygous *STS* deletion. Loss of *STS* enzyme activity leads to accumulation of cholesterol sulfate in the stratum corneum, disrupting epidermal barrier function and the normal desquamation process, resulting in the classic ichthyotic scaling seen in XLI (6, 8, 22). Beyond its role in epidermal lipid metabolism, *STS* is also a key enzyme in the metabolism of androgen precursors, mediating the conversion of DHEAS to dehydroepiandrosterone. This metabolic function explains the reduced DHEAS level seen in this patient, and highlights the systemic effects of *STS* deficiency beyond the skin. While XLI is primarily characterized by cutaneous manifestations, accumulating evidence indicates that *STS* deficiency is associated with an increased risk of neurodevelopmental alterations, including attention deficit hyperactivity disorder, autism spectrum traits, cardiac arrhythmia (23), and mild intellectual disability (9). It is important to distinguish the phenotypic spectrum of an isolated *STS* deletion from that of a larger Xp22.3 contiguous gene deletion syndrome. Most patients with XLI (90%) carry deletions limited to the *STS* gene, whereas some harbour larger deletions at Xp22.3 that encompass neighbouring genes. The Xp22.3 region harbours several clinically important genes in close proximity to *STS*, including *KAL1* (*ANOS1*), *NLGN4X*, and *SHOX* (24). When a deletion extends beyond *STS* to involve these neighbouring genes, the resulting composite phenotype reflects the additive effects of haploinsufficiency at multiple loci; such patients may present with intellectual disability and hypogonadotropic hypogonadism in addition to ichthyosis. In our patient, copy number variation analysis confirmed that the deletion is confined to exons 1–11 of *STS* and does not extend into the flanking *KAL1*, *NLGN4X*, or *SHOX* loci. Consistent with an isolated *STS* deletion, the patient had no cryptorchidism, anosmia, or hypogonadism. The patient’s mild intellectual disability and distinctive craniofacial dysmorphism are therefore more likely attributable to *KMT2B* haploinsufficiency and a possible additive effect of the *STS* deletion on cognition, rather than to an Xp22.3 contiguous gene deletion syndrome.

The patient’s complex multisystem presentation required careful consideration of several genetic syndromes with overlapping endocrine and cutaneous manifestations. McCune-Albright syndrome (MAS; OMIM:174800), classically defined by the triad of polyostotic fibrous dysplasia, café-au-lait skin pigmentation, and peripheral precocious puberty, was an important differential diagnosis (25). However, several clinical features distinguished this patient from MAS. The skin lesions were ichthyotic, dark-brown, polygonal scales predominantly affecting the neck and extensor surfaces of the lower limbs, consistent with XLI, rather than the café-au-lait macules with irregular borders characteristic of MAS. The precocious puberty was central (gonadotropin-dependent) in origin, as confirmed by a pubertal GnRH stimulation test, whereas MAS typically causes peripheral precocious puberty driven by autonomous gonadal activation (26). No clinical or radiographic evidence of fibrous dysplasia was present. Whole-exome sequencing did not identify any pathogenic variants in *GNAS*, which is responsible for MAS through postzygotic activating mutations (27). Neurofibromatosis type 1 was excluded on the basis of absent neurofibromas, Lisch nodules, and NF1 mutations. Kabuki syndrome, which shares phenotypic overlap with *KMT2B*-related disorders including intellectual disability and distinctive facial features, was distinguished by the absence of its characteristic facial gestalt, fetal fingertip pads, and skeletal anomalies, as well as by the absence of mutations in *KMT2D* or *KDM6A* (28). The definitive molecular diagnosis of a *de novo KMT2B* loss-of-function variant and a hemizygous *STS* deletion provided a complete genetic explanation for all of the patient’s clinical manifestations, eliminating the need for further diagnostic speculation.

The diagnostic approach used in this case illustrates key principles for the evaluation of children with complex, unexplained multisystem phenotypes. Targeted single-gene testing or phenotype-specific gene panels are often insufficient to identify concurrent molecular defects, particularly when one variant is a single nucleotide change and the other is a copy number alteration. Combined WES and CNV analysis enables simultaneous detection of both types of genomic variation, substantially improving diagnostic yield in patients with complex phenotypes (29). For patients with ichthyotic skin changes, particularly those with scaling predominantly affecting the neck and extensor surfaces of the limbs, targeted assessment of *STS* is warranted, even in the presence of other neurodevelopmental or endocrine features. Similarly, in patients with intellectual disability and distinctive craniofacial features, *KMT2B*-related disease should be included in the differential diagnosis even in the absence of dystonia, to avoid delayed or missed diagnosis.

Longitudinal multidisciplinary care is essential for optimizing outcomes in patients with digenic rare diseases. The management plan for this patient focused on three core clinical domains: suppression of precocious puberty using a GnRH analog to preserve adult height potential, supportive dermatological care to maintain skin integrity and quality of life, and prospective neurological monitoring to enable early intervention for dystonia should it develop. While there are currently no disease-modifying treatments for *KMT2B*-related disorders, supportive interventions, rehabilitation therapy, and surgical options such as deep brain stimulation are available for patients who develop severe, treatment-refractory dystonia (10).Genetic counseling is a critical component of care for this family, addressing the *de novo* nature of the *KMT2B* variant, which confers a low recurrence risk for siblings, and the XLI pattern of the *STS* deletion, which warrants carrier testing for the mother and discussion of prenatal diagnostic options for future pregnancies.

## Conclusion

This case documents the co−occurrence of a *KMT2B*−related disorder and XLI in a patient who presented with central precocious puberty, mild intellectual disability, and distinctive craniofacial features without dystonia to date. It demonstrates that CPP can be a presenting feature of *KMT2B*−related disease in patients without dystonia, and provides documentation of GnRH analogue therapy and structured endocrine follow−up for CPP in this population, which has not been previously described to our knowledge. The *de novo* KMT2B nonsense variant c.4213del (p.Leu1405Ter) is classified as pathogenic. Although the precise molecular mechanisms linking *KMT2B* dysfunction to premature activation of the hypothalamic−pituitary−gonadal axis remain to be fully defined, *KMT2B* haploinsufficiency may contribute to endocrine dysregulation through combined disruption of ERα−mediated transcriptional regulation and altered epigenetic programming at key hypothalamic developmental genes. Importantly, while the patient is currently dystonia−free, the variable age of onset in *KMT2B*−related disorders means delayed emergence remains possible, warranting ongoing surveillance.

These findings highlight the value of combined sequencing and copy number variation testing for timely and accurate diagnosis in children with unexplained multisystem involvement, and underscore the need for multidisciplinary follow−up incorporating endocrine, neurological, dermatological, and developmental surveillance.Future research should aim to elucidate how *KMT2B* dysfunction contributes to endocrine dysregulation and to clarify the potential additive effects of concurrent *KMT2B* and *STS* loss on neurodevelopmental outcomes.

## Data Availability

The original contributions presented in the study are included in the article/supplementary material. Further inquiries can be directed to the corresponding author.
