# Correction: Central precocious puberty as the initial manifestation of multisystem involvement caused by *de novo* heterozygous *KMT2B* mutation and STS hemizygous deletion: a case report

**DOI:** 10.3389/fendo.2026.1911609

**Published:** 2026-07-08

**Authors:** Yaqin Feng, Li Yang, Qing-Bo Xu, Lan-Fang Cao

**Affiliations:** Jiangxi Provincial Children’s Hospital; JXHC Key Laboratory of Precise Prevention and Treatment for Childhood Diabetes, Nanchang, Jiangxi, China

**Keywords:** *KMT2B*, *STS*, central precocious puberty, X-linked ichthyosis, pediatric endocrinology

In **Table 2** the reference for “Forzano F et al.” was erroneously written as “30”. It should be “31”.

In **Table 2** the reference for “Li XY et al.” was erroneously written as “31”. It should be “32”.

The reference range for inhibin B was incorrectly written as “27.06-8.96 pg/mL”.

A correction has been made to the “**Case report**” section, paragraph 3:

“inhibin B was 217.58 pg/mL (reference range: 27.06-48.96 pg/mL)”

The original version of this article has been updated.

**Table 2:** KMT2B-related cases with endocrine dysfunction (excluding isolated short stature).

Reference	Year	Phenotypic subgroup	Sex	Age at report	Mutation/Deletion	Endocrine manifestations	Precocious puberty (Y/N)	GnRHa treatment (Y/N)
Faundes V et al. (30)	2017	KMT2B-Related NDD	F	11y	c.1808dupC p.(Leu604Profs*72)	growth hormone deficiency	N	N
Ding S et al. (4)	2024	DYT-KMT2B	M	4y6m	c.8_9insGGCGGCG (p.Ala3Alafs*115)	thyroid dysfunction	N	N
Ding S et al. (4)	2024	DYT-KMT2B	F	15y4m	c.3605dupT (p.Met1202Ilefs*22)	earlier breast development and menstruation, advanced bone age	Y	N
Ding S et al. (4)	2024	DYT-KMT2B	F	20y9m	c.4214dupT (p.Ser1406Glufs*272)	Short stature, advanced bone age,earlier menstruation	Y	N
Cif et al. (5)	2020	DYT-KMT2B	F	22y	c.12_24dup13(p.Ser9Glyfs*111)	Precocious puberty (8 y)	Y	N
Cif et al. (5)	2020	DYT-KMT2B	M	19y	c.188delG(p.Ala40Profs*6)	Precocious puberty (9y)	Y	N
Cif et al. (5)	2022	DYT-KMT2B	M	19y	c.188delG(p.Ala40Profs*6)	Precocious puberty (9y)	Y	N
Cif et al. (5)	2020	DYT-KMT2B	M	26y	c.1107dupC(p.Glu370Argfs*19)	insulin dependent diabetes mellitus,Hypothyroidism	N	N
Cif et al. (5)	2020	DYT-KMT2B	F	29y	c.2137dupA(p.Thr713Asnfs*4)	Precocious puberty	Y	N
Cif et al. (5)	2020	DYT-KMT2B	F	9y	c.3602delC(p.Pro1201Argfs*154)	Precocious puberty	Y	N
Cif et al. (5)	2020	DYT-KMT2B	M	17y	c.3997delG(p.Glu1333Argfs*22)	Precocious puberty,Hypothyroidism	Y	N
Cif et al. (5)	2020	DYT-KMT2B	M	11y	c.4931G>T(p.Cys1644Phe)	Precocious Puberty	Y	N
Cif et al. (5)	2020	DYT-KMT2B	F	44y	c.6439C>T(p.Gln2147)	Hypothyroidism	N	N
Cif et al. (5)	2020	DYT-KMT2B	M	11y6m	c.7348 C>T(p.Arg2450)	Precocious puberty	Y	N
Forzano F et al. (31)	2012	19q13 microdeletion(KMT2B-Related NDD)	F	6y6m	a maternally inherited 3.32 Mbdeletion of chromosome 8q24.21e24.22, a de novo 1.70 Mb deletion of chromo-some 18q22 anda de novo 1.37 Mb deletion of the chromosome 19q13.11e13.12.	Primary hypothyroidism, GH deficiency, partial ACTH deficiency	N	N
Li XY et al. (32)	2020	DYT-KMT2B	F	11y	c.3605dup (p.Met1202Ilefs*22)	earlier breast development and menstruation, advanced bone age	Y	N
Indelicato E et al. (10)	2026	DYT-KMT2B	M	26y	c.2941T>C (p.Cys981Arg)	History of growth hormone therapy	N	N
Present case	2026	Non−dystonic KMT2B−related disorder	M	8y10m	c.4213del (p.Leu1405Ter)	CPP, marginally low FT4	Y	Y

DYT−*KMT2B*, *KMT2B*−related dystonia; *KMT2B*−NDD, *KMT2B*−related neurodevelopmental disorder; CPP, central precocious puberty; GnRHa, gonadotropin−releasing hormone analog; GH, growth hormone; ACTH, adrenocorticotropic hormone; FT4, free thyroxine; M, male; F, female; y, years; m, months.

Isolated short stature, which is a common but nonspecific finding in *KMT2B*−related disorders, was not included in this comparison unless accompanied by other endocrine abnormalities. The present case appears in bold.Non−dystonic *KMT2B*−related disorder

